# Strangulated inguinal indirect hernia as a cause of secondary torsion of the greater omentum: A rare case report and literature review

**DOI:** 10.1097/MD.0000000000042511

**Published:** 2025-07-25

**Authors:** Hong-yi Chen, Zi-peng Huang, Bin Zu, Long-zhi Zheng

**Affiliations:** a The Third Clinical Medical College, Fujian Medical University, Fuzhou, Fujian Province, People’s Republic of China; b Department of Gastrointestinal Surgery, The Affiliated Hospital of Putian University, Putian, Fujian Province, People’s Republic of China.

**Keywords:** acute abdomen, diagnosis, incarcerated hernia, omental torsion, treatment

## Abstract

**Rationale:**

Omental torsion is a rare surgical acute abdomen. Due to the lack of specific clinical manifestations and atypical symptoms, preoperative diagnosis is very difficult, and it is often confused with acute abdomen caused by other reasons. Secondary omental torsion caused by the incarcerated hernia is extremely rare.

**Patient concerns:**

A 40-year-old male patient was admitted to the hospital on January 1, 2020, due to “nonrecoverable mass in the right inguinal region for 6 hours.” The patient had a history of right inguinal hernia for 5 years and denied a history of abdominal trauma or surgery. Physical examination: normal vital signs, slightly distension in the abdomen, light tenderness in the whole abdomen, obvious in the right lower abdomen, accompanied by light rebound tenderness, weak bowel sounds. A mass of about 5 cm × 5 cm × 6 cm can be touched in the right inguinal region, with medium texture and obvious tenderness.

**Diagnoses:**

Secondary omental torsion caused by an incarcerated inguinal hernia on the right side.

**Interventions:**

Laparoscopic exploration.

**Outcomes:**

The patient recovered well and was discharged on the 8th day after surgery.

**Lessons:**

Omentum torsion is a rare disease that is difficult to diagnose preoperatively, and surgeons should raise awareness of it. Detailed medical history consultation, careful physical examination, combined with auxiliary examination, can help to reduce misdiagnosis.

## 1. Introduction

Omental torsion refers to the torsion of part or all of the greater omentum centered on the longer axis of the omentum, which usually rotates clockwise by 360° to 720°, forming an irreversible state, which can lead to ischemia and necrosis of the distal tissues. Omental torsion is a rare surgical emergency, with an incidence of only 0.18% reported by a single center.^[1]^ This disease is commonly seen in young and middle-aged people, mostly in the age group of 30 to 50 years old, mostly in males, rarely in children and the elderly, and is more common in males than females, with a male-to-female ratio of about 2:1.^[2]^ Omental torsion can cause obvious abdominal pain and gastrointestinal symptoms, and it is easy to be misdiagnosed as appendicitis, cholecystitis, and other acute abdominal diseases. Preoperative diagnosis is rarely clear, and the diagnosis is usually made after abdominal exploration. This paper retrospectively analyzes a case of secondary omental torsion caused by a right-incarcerated indirect inguinal hernia in our hospital, and reviews relevant literature to explore the clinical diagnosis and treatment of omental torsion.

## 2. Case report

**Figure 1.**
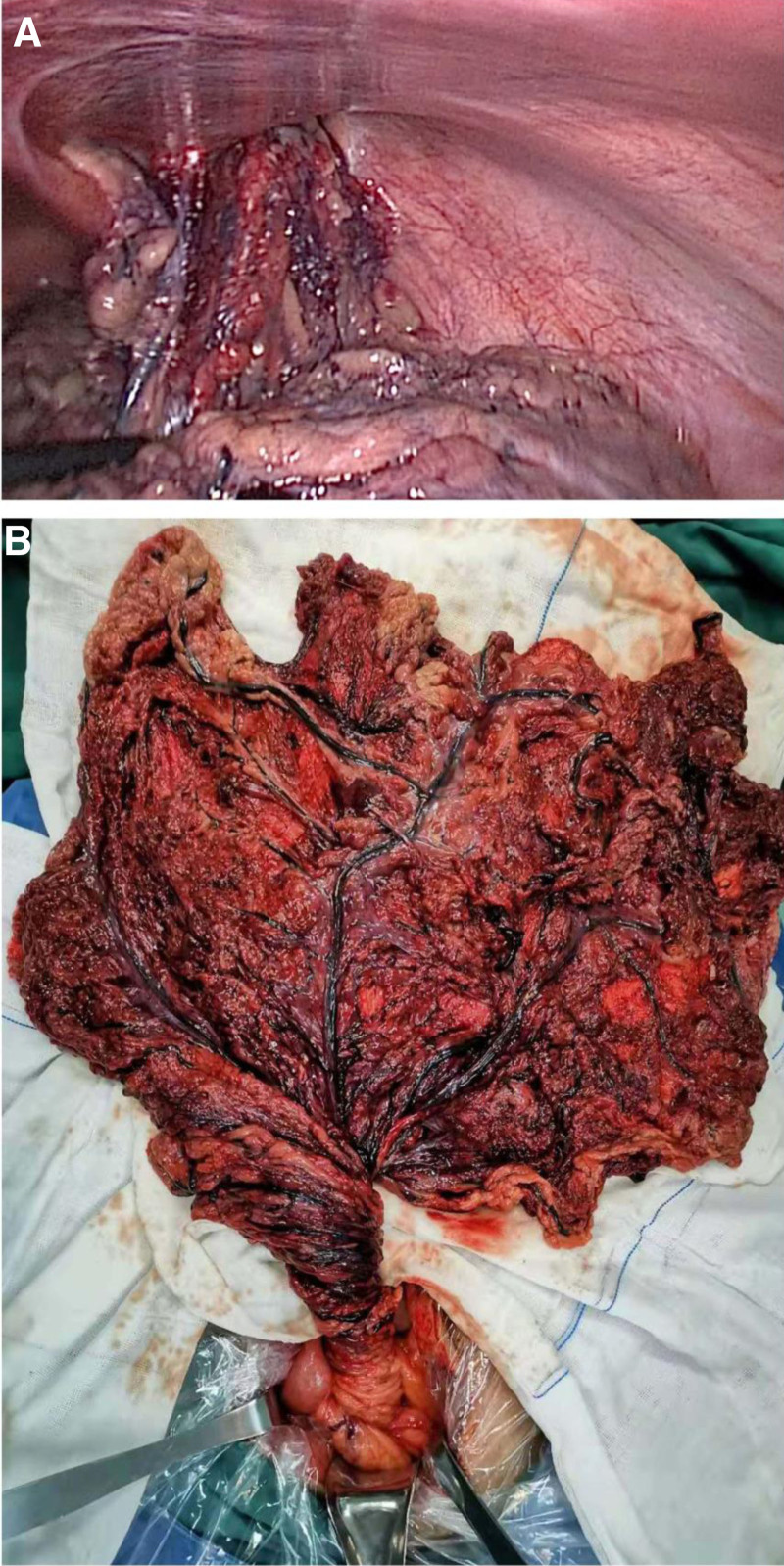
Omentum incarcerated in the right inguinal canal (A). The omentum was rotated 720° clockwise from the root with ischemic necrosis (B). The picture was taken during the emergency surgery.

A 40-year-old male patient was admitted to the hospital on January 1, 2020, due to “nonrecoverable mass in the right inguinal region for 6 hours.” The patient had a history of right inguinal hernia for 5 years and denied a history of abdominal trauma or surgery. Physical examination: normal vital signs, slightly distension in the abdomen, light tenderness in the whole abdomen, obvious in the right lower abdomen, accompanied by light rebound tenderness, and weak bowel sounds. A mass of about 5 cm × 5 cm × 6 cm can be touched in the right inguinal region, with medium texture and obvious tenderness. Manipulative reduction was unsuccessful. After admission, abdominal computed tomography (CT) scan showed a right indirect inguinal hernia and mesenteric lesions in the middle and lower abdomen, and mesenteric panniculitis was considered. The patient was diagnosed with a right strangulated inguinal indirect hernia, and then laparoscopic exploration was performed. Intraoperatively, the red bloody ascites in the abdominal cavity was about 100 mL, the right inguinal inner ring was enlarged with a diameter of about 3 cm, and the hernia contents were omentum. Moreover, the omentum was rotated clockwise from the root to about 720°, accompanied by ischemia and necrosis (Fig. 1). The inner ring of the right inguinal hernia was repaired by pouch-string suture, and the omentum was excised. The patient recovered well and was discharged on the 8th day after surgery.

## 3. Discussion

Omental torsion is a very rare acute abdomen. The symptoms are similar to many acute abdomen conditions and are rarely diagnosed definitely before surgery. It has been reported that only 0.2% to 4.8% of all clinical cases of omental torsion can be correctly diagnosed before surgery.^[3]^ Eitel first described omental torsion in 1899.^[4]^ Omental torsion infarction is a very rare acute abdominal disease, with only about 300 cases have been reported in the world,^[5]^ and omental torsion caused by incarcerated inguinal hernia is even rarer.

Omental torsion can be classified as primary and secondary according to the causes of its occurrence. The cause of primary omental torsion is still unclear, but it is generally believed to be related to the abnormal anatomical morphology of the great omentum (such as hyperelongation of omentum and abnormal blood supply vessels), the change of position (such as strenuous exercise and sudden change of body position), and the change of intra-abdominal pressure (increased intra-abdominal pressure).^[6]^ Secondary omental torsion is more common than primary omental torsion, and is often associated with intra-abdominal inflammation or the formation of strip adhesion bands after surgery, leading to the greater omentum torsion during exercise; as well as omental tumors, inguinal hernias, etc., which makes the omentum asymmetrical and easy to twist.

Omental torsion is a rare surgical acute abdomen with low incidence and atypical clinical presentation. The symptoms are similar to many acute abdomens. The common symptoms are abdominal pain and gastrointestinal symptoms, manifested as persistent nonradioactive pain, and the pain tends to worsen, often cannot be relieved by drugs, accompanied by fever, nausea, vomiting, and other gastrointestinal symptoms. These manifestations are nonspecific, so the diagnosis of omental torsion is difficult, and it is easy to be misdiagnosed as appendicitis, cholecystitis, diverticulitis, and other acute abdominal diseases clinically. The preoperative diagnostic rate is low, and surgical exploration is often required to make a definite diagnosis. Goti et al reported that 66% of the cases presented as acute appendicitis and 22% presented as cholecystitis.^[7]^

At present, there is no specific examination method for the torsion of the omentum, and conventional examination methods such as X-ray and ultrasound have little diagnostic significance, while abdominal CT and magnetic resonance imaging are helpful for the diagnosis of omental torsion. The typical CT manifestation of omental torsion is whirlpool sign, which has a certain value for clinical diagnosis, but it is not specific for the diagnosis of omental torsion. Because not all omental torsion has the typical whirlpool sign, it is difficult to distinguish it from other omental diseases, especially segmental omental infarction and primary epiploic appendagitis. In our case of omental torsion, the CT scan of the radiologist was considered mesenteric panniculitis. In addition, omental torsion is relatively rare in clinical practice, and physicians do not have enough understanding of the signs of greater omentum torsion, which may easily lead to misdiagnosis. Therefore, the diagnosis of greater omentum torsion is often made clear after abdominal exploration.

The final diagnosis of omental torsion requires laparotomy. With the development of minimally invasive technology, laparoscopic exploration can be used as both an examination method and a surgical treatment method, with dual advantages of diagnosis and treatment. And, this kind of surgery is more consistent with the concept of enhanced recovery after surgery, reducing surgical stress, maintaining a stable internal environment, speeding up postoperative rehabilitation, shortening hospital stay, and reducing hospitalization costs. Therefore, laparoscopy is the preferred method for the diagnosis and treatment of acute omentum torsion. After the diagnosis of omentum torsion is confirmed, resection is usually performed above 2 cm of necrotic omentum. If the diagnosis of omentum torsion due to incarcerated inguinal hernia is considered, an exploratory laparotomy should be performed instead of an inguinal incision because it is possible to avoid the missed diagnosis of ischemic necrosis of the greater omentum. Whether to perform tension-free hernioplasty at the same time depends on the patient’s general condition and the degree of local contamination.

## 4. Conclusion

In summary, omentum torsion is a rare disease that is difficult to diagnose preoperatively, and surgeons should raise awareness of it. Detailed medical history consultation, careful physical examination, combined with auxiliary examination, can help to reduce misdiagnosis. If the diagnosis is unclear, or the patient’s symptoms persist or even worsen, surgery should be performed. Laparoscopic surgery has great advantages, and the surgical method is laparoscopic or open resection of necrotic greater omentum. For omental torsion caused by inguinal hernia, clinicians should not be satisfied with the diagnosis and treatment of incarcerated hernia alone. For patients with obvious abdominal signs, laparoscopic exploration is recommended because the traditional inguinal incision cannot adequately meet the needs of abdominal exploration.

## Author contributions

**Writing – original draft:** Hong-yi Chen.

**Investigation:** Zi- peng Huang, Bin Zu.

**Writing – review & editing:** Long-zhi Zheng.
